# Impact of root canal shaping using TruNatomy on postoperative pain and operative torque generation: a randomized clinical trial

**DOI:** 10.1186/s12903-025-06418-z

**Published:** 2025-07-19

**Authors:** Tarek Ali Al-Mosalmy, Heba Maged El-Far, Madiha Mahmoud Gomaa, Dina Ahmed Morsy

**Affiliations:** 1 Cairo University, Faculty of Dentistry, Department of Endodontics, Cairo, Egypt; 2 Misr International University, Faculty of Dentistry, Obour City, Egypt

**Keywords:** Endodontics, Necrotic teeth, Operative torque, Post operative pain, TruNatomy

## Abstract

**Background:**

TruNatomy instruments promises a more conservative alternative to conventionally used instruments. They are claimed by the manufacture to decrease the amount of debris extrusion as well as the torque generated during instrumentation, potentially resulting in lower post-operative discomfort and a safer more convenient experience for the patient and clinician.

**Aim:**

To compare the effect of canal shaping using TruNatomy (TN) rotary system to RaCe (RC) rotary system on post-operative pain in necrotic maxillary bi-rooted premolars, while simultaneously inspecting the real-time dynamic forces produced during instrumentation.

**Methods:**

In this parallel group superiority trial, following power calculation, 40 patients diagnosed with pulp necrosis in asymptomatic bi-rooted maxillary premolars were recruited and randomly allocated into two equal groups (*n* = 20) according to the instrumentation system used. Treatment was completed in a one visit for all cases. Pain levels were recorded immediately before RCT and at 6,12,24,48, 72 h, and 1-week post RCT using mVAS. The number of analgesics taken if any was also recorded. Operative torque generated during root canal preparation as well as the time taken for the instrument to reach the working length were simultaneously recorded during instrumentation. Outcome data was statistically analyzed using Shapiro Wilk test, independent* t* test, Mann–Whitney U, Chi square, Fisher exact tests, spearman’s correlation coefficient. Significance level (α) was set at 0.05.

**Results:**

There was no statistically significant difference between pain scores in the test groups at all time points measured (P > .05). No significant different was also recorded in terms of number of analgesics taken (P > .05). Mean torque, average peak torque and maximum torque values showed no statistically significant difference as well, however the instrumentation time was significantly shorter for the TN Group (P < .05). A moderate significant positive correlation was found between the instrumentation time and generated operative torque.

**Conclusion:**

TN and RC rotary instruments resulted in similar and acceptable levels of post-operative pain in cases of asymptomatic necrotic teeth. However, TN rotary system combined torque and instrumentation time values suggest an overall higher cutting efficiency and potentially a better safety profile as compared to RC rotary system.

**Trial registration:**

ClinicalTrials.gov identifier: NCT04616469; registration date (10/08/2020).

## Background

Pain is an unfortunate but common consequence of root canal treatment procedures. Previous studies examining the occurrence of post-operative pain have found it to range between 25- 40% within the first 24h [1, 2]. This was reported to decrease progressively to approximately 11% by the end of the first week following treatment [3]. While pain can be an unpreventable consequence of root canal treatment, various factors have been suggested to influence its occurrence. These include pre-operative factors such as age; gender; tooth type; pulp and periapical status, and pre-treatment pain levels, which are mostly unmodifiable factors. On the other hand, intra-operative and treatment-related factors such as number of visits, the method of root canal irrigation and activation, instrumentation technique, and type of instrument and obturation technique used are mostly modifiable factors. Accordingly, adjustment of these factors may play a beneficial role in the overall goal of reducing post-operative pain [4, 5].

Separation of endodontic instruments during use is another problem that may hinder accessibility for disinfecting the root canal system thus indirectly impacting the treatment outcome as well as the patient’s post-operative experience. Conventionally, the safety and durability of endodontic instruments are tested using static laboratory tests such as the flexural cyclic fatigue and torsional resistance tests. However, these methods were found to be plagued with two inherent flaws: the concentration of load at specific parts of the instrument and the static conditions at which these tests are performed where each stress condition is isolated and separately examined [6]. While these stress conditions can be isolated in laboratory static conditions, clinically the instrument is subjected to a multitude of stresses simultaneously in a more dynamic fashion. Accordingly, a need for a more representative testing method for instrument performance and interactions in clinical situations remains a concern [7].

Thanks to recent advances in endodontic motors and software technology the torque generated during use and the time taken by the instrument to reach the working length (WL) can be recorded by the endodontic motor and used as an indicator for instrument's performance in terms of its cutting efficiency, expected durability, and overall safety [8]. This method of evaluation has the advantage of a more accurate replication of the clinical conditions where instruments are subjected to an assimilation of forces distributed across their entire length simultaneously. Furthermore, it can be correlated with its in-vitro performance to better categorize the potential safety profile of the instrument utilizing the torque range parameter introduced by *Di Nardo *et al*. (2020)* [6]. While some studies have examined the prospective applications of the operative torque parameter, its potential is yet to be fully explored [9].

In this regard, instrument design elements such as its core-size, taper, and cross-sectional design, can play a significant role in its clinical behavior. Accordingly, adjustment of these elements may impact the stress levels placed on the instrument, its ability to evacuate and prevent apical extrusion of debris, as well as the time it takes to complete the instrumentation process [10, 11]. This, in turn, can have either a positive or negative impact on the durability and safety profile of the instrument as well as the patient’s post-operative pain experience.

TruNatomy (TN) is a novel instrument made from special nickel titanium heat-treated wire and presented with an innovative new design. Its slim shape, unique cross-section, and off-centred design are professed to provide greater shaping efficiency with less torque and shorter working time. Moreover, its unique design is claimed to provide more space for debris escape thus subsequently leading to less post operative pain. The impact of the instrument design on the post-operative pain and as well as operative torque and time have not been subjected to adequate scrutiny.

Accordingly, this study aimed to examine the effect of canal shaping using TN on postoperative pain in necrotic maxillary bi-rooted premolars in comparison to RaCe (RC) rotary system while simultaneously employing the parameter of operative torque for the examination of the resultant real-time dynamic forces produced during instrumentation.

## Methods

### Protocol registration and study design

Patients and statisticians were blinded to the interventions in this single-center, parallel, prospective, randomized, superiority clinical trial, which had a 1:1 allocation ratio. One endodontist who was familiar with both systems completed all therapeutic procedures in a single visit. Clinicaltrials.gov was used for registering the study protocol (ClinicalTrials.gov identifier: NCT04616469); registration date (10/08/2020). The Consolidated Standards of Reporting Trials (CONSORT) were followed in the writing of this randomized clinical trial [12].

### Ethics approval and consent to participate

The protocol and informed consent forms were examined and approved by the ethical committee of the Faculty of Dentistry, Cairo university for their scientific content and adherence to applicable research and human subjects' regulations (approval code: 32:10:20). The methods used in this study were in compliance with the 2013 revision of the World Medical Association's Declaration of Helsinki. The procedure along with its associated benefits and risk were thorough explained to the patients and an informed consent for trial participation was obtained from all patients involved in the study.

### Participants selection

Patients who met the inclusion criteria were enrolled from the outpatient clinic of the Department of Endodontic after approving to participate in the trial and signing the informed consent.

### Eligibility criteria

#### Inclusion criteria

- Systematically healthy patients ASA class I or II according to the American Society of Anesthesiologists)
- Males and females
- Maxillary bi-rooted premolar diagnosed clinically with pulp necrosis, possessing two separate fully formed roots each having type I patent canal, with a tooth length ranging between 18-22 mm, and no or slight widening of PDL space.

#### Exclusion criteria

- Patients who received analgesic in the past 12 h
- Patients with active periodontal disease
- Badly destructed teeth
- Teeth with moderate to severely curved roots (> 5°)
- Teeth with canals initially larger than size #15 k-file.

### Sample size calculation

In order to conduct a two-sided statistical test of the null hypothesis that there is no difference in post-operative pain between TN and RC rotary systems a power analysis of adequate power was conducted. The anticipated sample size (n) was a total of 34 instances by using an alpha (α) level of 0.05 (5%), a beta (β) level of 0.20 (20%), i.e., power = 80%, and an effect size of (d = 1) based on the findings of (Zand et al., 2016) [10] and expert opinion. To account for potential dropouts during follow-up periods, the sample size was raised by 15% to 40 cases (*n* = 20 cases per group). G*Power 3.1.9.4 was utilized to calculate the sample size.

### Random sequence generation, allocation concealment and blinding

Random sequence generation was performed by an independent third party not involved in the patient enrolment using GraphPad Software (Boston, MA). The sequence generated was kept hidden from the operator. Sequentially numbered opaque sealed envelopes denotating either the intervention or control which were handed to the operator following access cavity preparation. The operator could not be blinded to the instrument used, however the participants which were also the outcome assessor were blinded to the study’s intervention. The statistician was also blinded to the group’s assignment.

### Diagnostic procedures

Patients presenting with asymptomatic maxillary bi-rooted premolars teeth diagnosed with pulp necrosis discovered during routine examination were recruited for the study based on the previously mentioned criteria. A final diagnosis of Pulp necrosis was confirmed by the lack of bleeding following access cavity preparation.

### Endodontic procedures

**Fig. 1:**
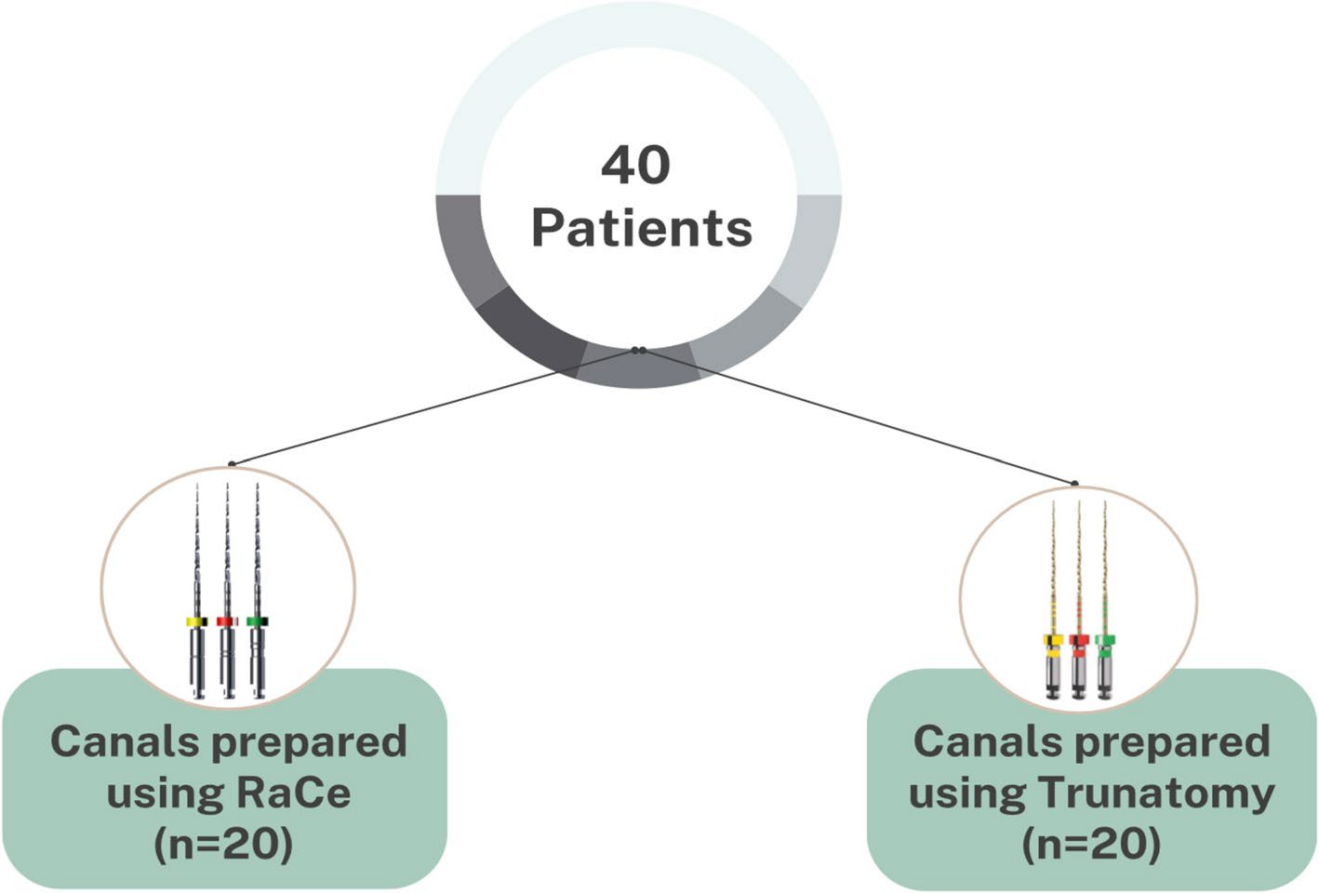
Study groups allocation

Treatment of all cases was done in a one visit. Infiltration technique using 1.8 ml 4% Articaine hydrochloride (epinephrine 1:100000) (Septodont Saint Maur des-Fossés, France) with a 27-gauge short needle was used. The tooth was isolated and caries and/or coronal restoration were completely removed using a sterile round bur. Access cavity completed using carbide round bur size #2 and Endo-Z bur. Copious irrigation of the access cavity was performed using 3 ml of 5.25% NaOCl. Patency of the canals was checked using a size #10 K-file with the aid of an electronic apex locator (Root ZX, J. Morita USA, Irvine, CA.) Tooth length was then established using the electronic apex locator and confirmed with one or more periapical radiographs. Glide path was created using size #10 and #15 K-file. Patients were then randomly allocated to the study groups according to the randomization protocol (Fig. 1).

Group A: Canals were prepared using TN rotary instruments in the following sequence: Small (#20 variable taper 0.04), Prime (#26 variable taper 0.04) and Medium (#36 variable taper 0.03).

Group B: Canals were prepared using RC rotary instruments in the following sequence: #20/0.04, #25/0.04, and #35/0.04.

All instruments were used in accordance with the manufacturer’s instructions with a maximum torque of 1.5 Ncm and a rotation speed of 500 RPM for TN and 600 RPM for RC, powered by X-Smart IQ cordless endodontic motor. Shaping was done with an inward pecking motion (short amplitude and intermittent progression of the file 1–2 mm at a time) and slight apical pressure, up to the WL. The measured operative torque and time of preparation were automatically and simultaneously transferred and recorded into a dedicated software application (Endo IQ iPad App. Dentsply Sirona Endodontics US, CA) connected to the endodontic motor (X-Smart IQ. Dentsply Sirona Endodontics US, CA).

The canals were thoroughly irrigated using 3 ml of 2.6% NaOCl between every subsequent instrument exchange using 30-gauge side vented needle (Steri irrigation tips; Diadent, Chungcheongbuk-do, Korea.), 1–2 mm shorter than the WL. A final rinse of 1 ml of 17% EDTA was performed for 1 min for each canal as a final flush and separated from the NaOCl using 5 ml of saline solution.

After taking a master cone-fit radiograph, the canals were dried with sterile paper points and obturated using a modified single-cone technique and resin-based sealer. Coltosol F temporary filler was used to close the access cavity.

Patients were directed to accurately document their pain levels on the provided modified VAS chart at 6- 12- 24- 48- hours and 7 days. Ibuprofen 400 mg was prescribed in case of severe pain and patients were asked to record any analgesics intake. Patients were referred for final restorations one week later.

#### Outcome measures

- 1- Postoperative pain assessment

Patients were instructed to record their post-operative pain levels using a 10 cm mVAS pain dairy where 0 = No pain, > 0 mm and < 20 mm = Mild pain, > 20 mm and < 40 mm = Moderate pain, > 40 mm and < 60 mm = Severe pain, > 60 mm and < 80 mm. = Very severe pain, > 80 mm and < 100 = The worst pain possible [13]. Pain levels were recorded at 6,12,24,48,72 h, and 1-week postoperatively and charts were submitted at the 2nd appointment after 1-week.

- 2- Analgesic intake assessment:

Analgesic tablets intake was measured at the end of the 1-week follow-up period. The patients were instructed to take ibuprofen 400 mg in the event of severe pain.

**Fig. 2:**
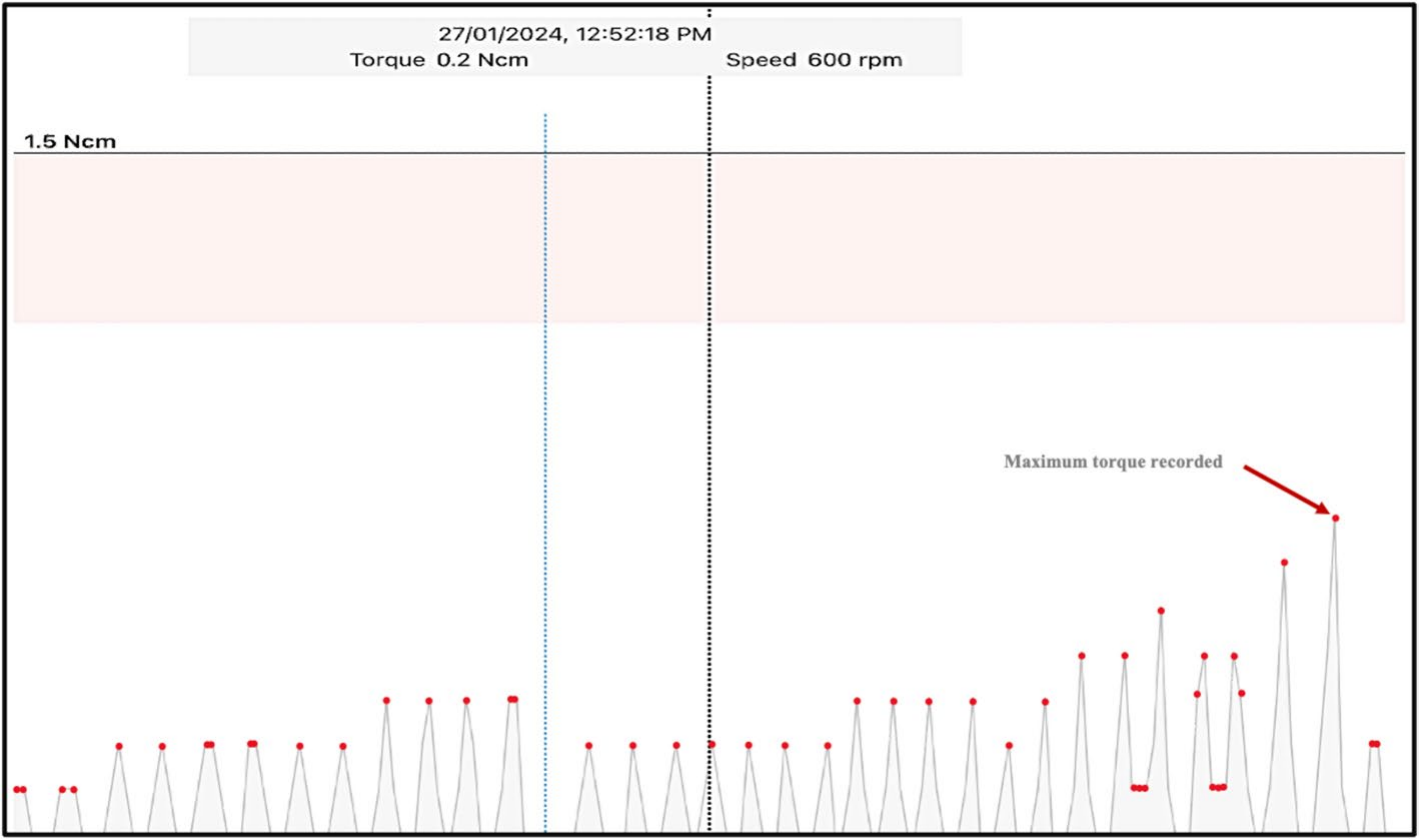
Operative torque graph showing average peak torque (Red Dots) and maximum torque reached (Red Arrow)

- 3- Real-time operative torque, Mean torque, average peak torque assessment:

The Real-time operative torque was measured throughout the instrumentation process using X-Smart IQ motor. Msean torque ( the average of all values registered during the motor running time), average peak torque (Mean values of positive torque peak points on the torque graph) as well as the maximum torque reached were extracted from the device and recorded on Excel spread sheet for analysis (Fig. 2).

- 4- Instrumentation time

The time needed for the instruments to reach the full WL was simultaneously recorded using the same endodontic motor from the point of insertion into the canal orifice and until the file reach the full WL. No changes to this study were necessary during data collection.

### Statistical analysis

The Shapiro–Wilk test was used to determine whether the data were normal. The mean, standard deviation (SD), median, minimum, and maximum values were used to display continuous data. When comparing unrelated samples of regularly distributed data, the independent t-test was employed, and when comparing unrelated samples of non-normally distributed data, the Mann–Whitney U test was employed. Frequencies (N) and percentages (%) were used to depict the categorical data, and the Chi-square or Fisher exact tests were used for analysis. For primary tests, a significance limit of p < 0.05 was established. The statistical analysis was conducted using IBM Corp.'s SPSS program, which was released in 2017. Armonk, NY: IBM SPSS Statistics for Windows, Version 25.0.

## Results

**Fig. 3:**
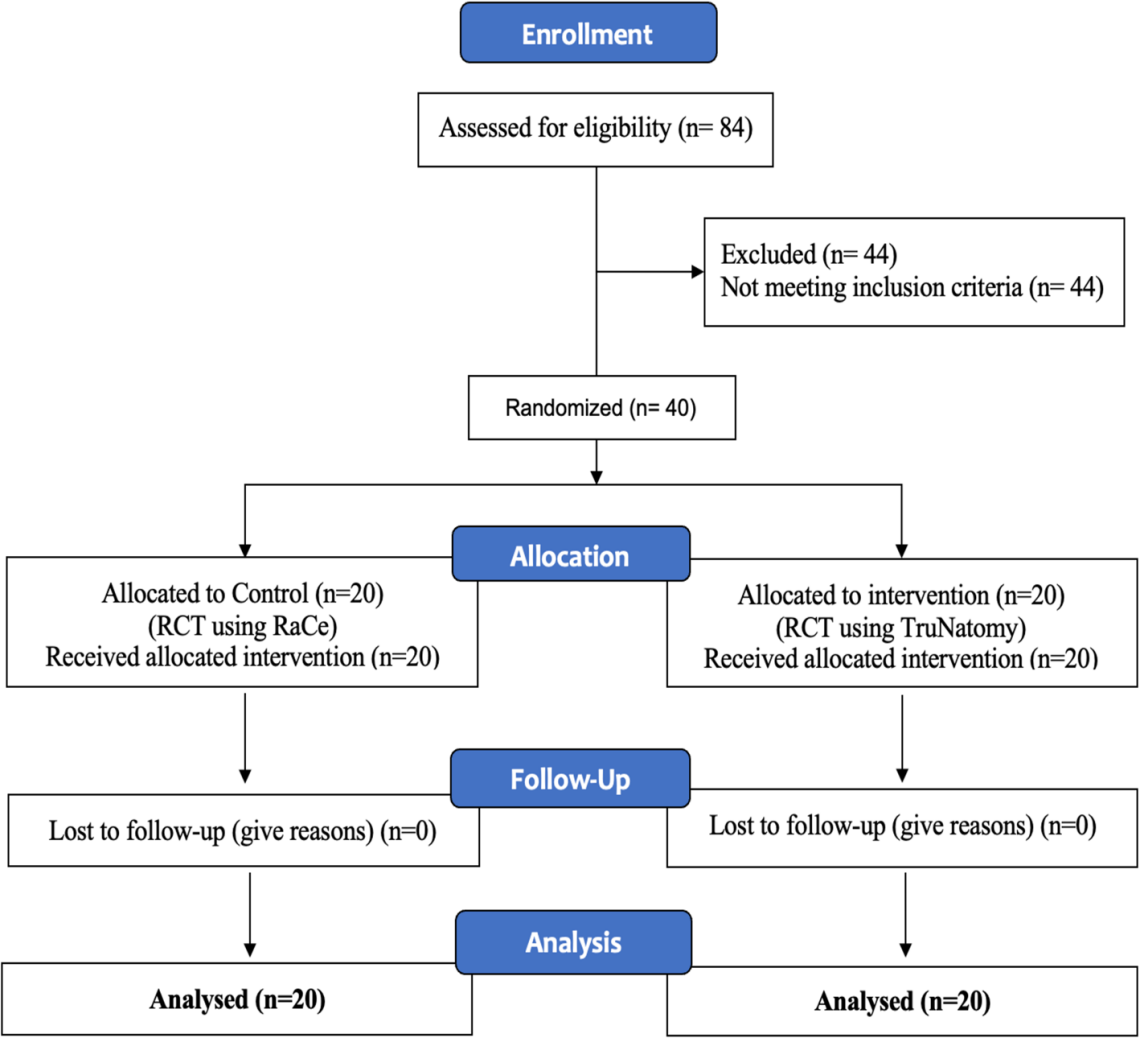
CONSORT diagram showing the participants flow during different stages of the trial

All patients completed the 1-week follow-up and returned for the final restoration with no loss to follow-up and all 40 patients were included in the analysis. Patients were recruited between the 1st of May 2023 and the 1st of July 2024. The enrollment procedure for patients and their progress through every phase of the study are shown in the CONSORT Flowchart (Fig. 3). Demographic data, descriptive statistics are presented in (Table 1).

**Table 1:** Frequencies, distribution, and the result of independent *t* test and Chi square test for comparison of age and gender distribution between the two groups

**Variables**		**Demographic data**				
		**TruNatomy**		**RaCe**		***p-*value**
		**Mean SD**		**Mean SD**		
**Age**		**38.1**	**(11.5)**	**38.2**	**(11.6)**	**0.989**
		**N**	**%**	**N**	**%**	
**Gender**	**Males**	**9**	**45.0%**	**10**	**50.0%**	**0.752**
	**Females**	**11**	**55.0%**	**10**	**50.0%**	

^*^Significant at p = *0.05*

**Fig. 4:**
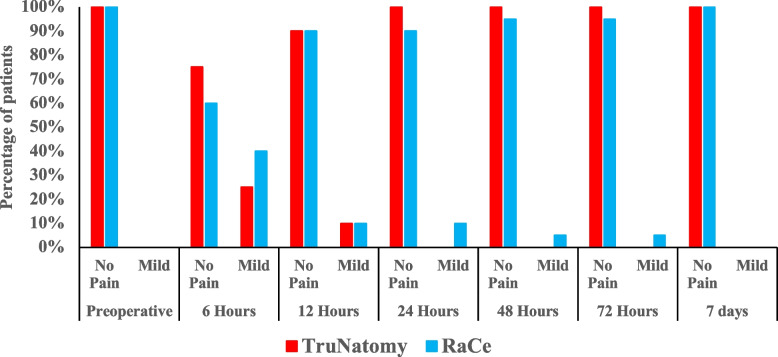
Bar chart representing the incidence of different pain categories at different time intervals in the two groups

**Fig. 5:**
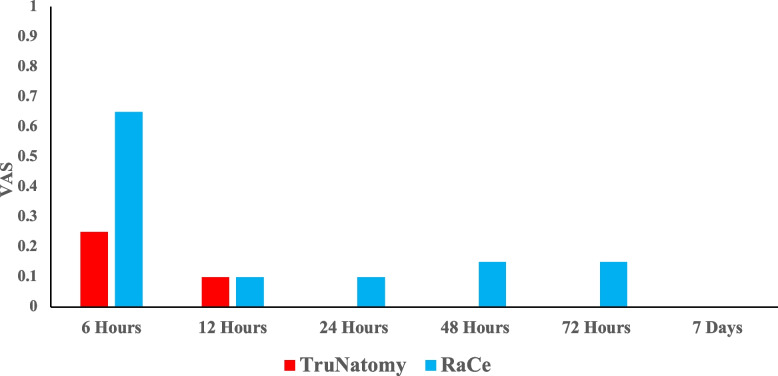
Bar chart representing the mean postoperative pain intensity at different time intervals within the two groups

**Fig. 6:**
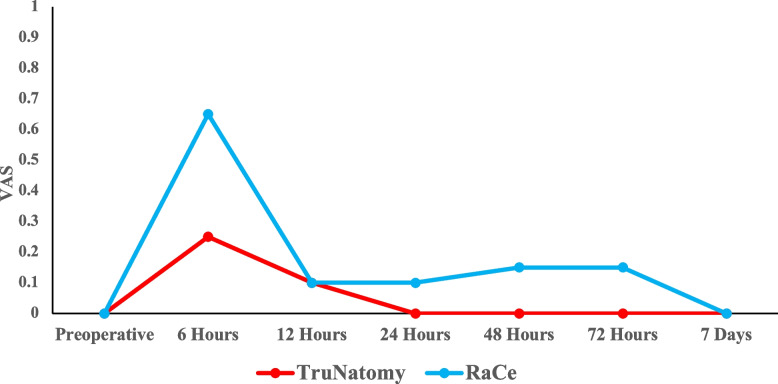
Line chart representing the changes in the mean pain intensity at different time intervals within each group

All patient presented no to mild pain (Fig. 4). There was no significant difference in terms of pain incidence between the two groups at all time points measured (P > 0.05). The mean pain scores are represented in (Fig. 5). The mean pain scores were lower in the TN group in comparison to the RC group at all the time points measured. However, there were no significant difference between the groups at any interval (P > 0.05). Intra-group comparisons revealed that pain levels were at its highest at the 6 h’ time point (*p* = 0.01) and decreased gradually where no pain was reported in both groups at the one-week interval (Fig. 6).

None of the patients enrolled required analgesics during the follow up period with no significant difference between the groups (*p* = 1.0).

**Fig. 7:**
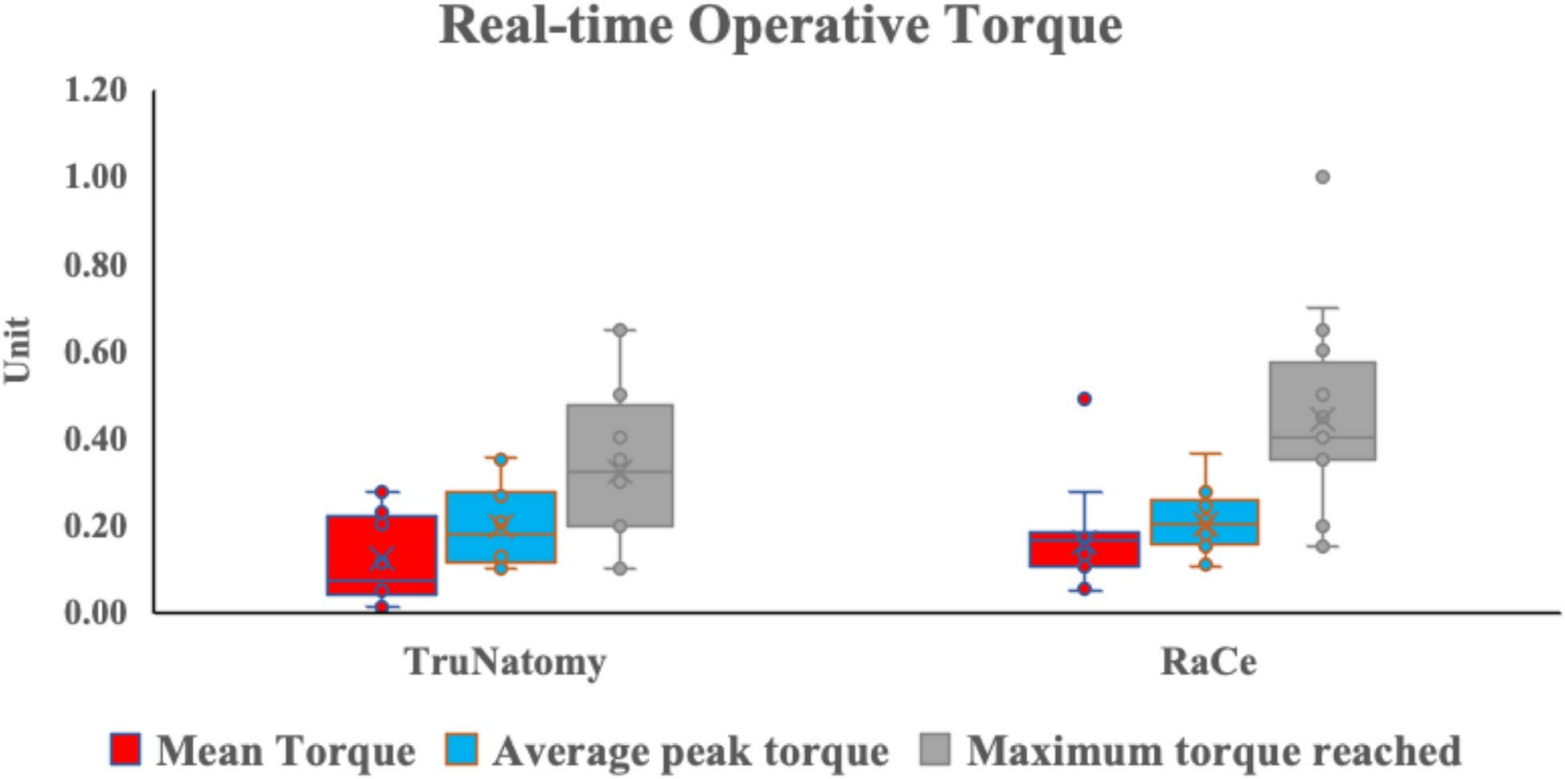
Box and whisker plot representing the median and interquartile range of mean torque, average peak torque, and maximum torque reached values in the two groups

For the mean operative torque TN rotary files trended toward lower torque values with median and range values of 0.07 (0.01—0.28) Ncm in the TN group and median and range values of 0.17 (0.05—0.49) Ncm in the RC group. A mean value of 0.2(0.1)Ncm was measured for the TN group in comparison to RC rotary files with a mean value of 0.2 (0.1)Ncm. There was no significant difference between the two groups (*p* = 0.398). (Fig. 7).

The average peak torque showed similar results with TN group reporting a median and range values of 0.18 (0.1—0.36) Ncm. while in the RC group a median and range of 0.20 (0.11—0.37) Ncm. The mean average peak torque was 0.2 (0.1) Ncm for the TN group and 0.2 (0.1) Ncm for the RC group. There was no significant difference between the groups (*p* = 0.799). (Fig. 7).

The median and range for the maximum torque reached for the TN group was 0.33 (0.10—0.65) Ncm and for the RC group it was 0.40 (0.15 – 1.0) Ncm. The mean and standard deviation values of the overall maximum torque were 0.3 (0.2) Ncm in the TN group and 0.4 (0.2) Ncm in the RC group. There was no significant difference between the two groups (*p* = 0.068). (Fig. 7).

**Fig. 8:**
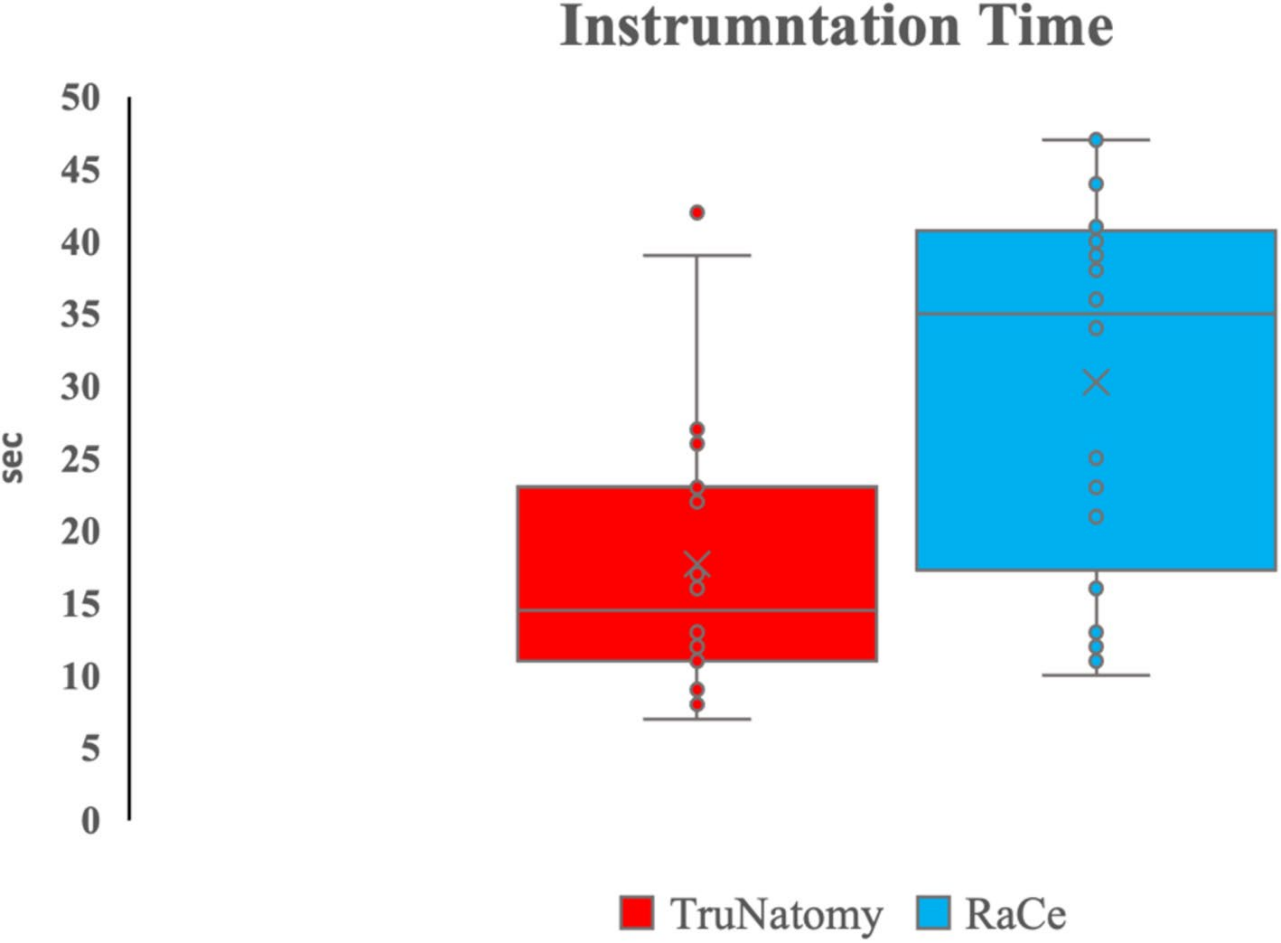
Box and whisker plot representing the median and interquartile range of the mean values of instrumentation time in the two groups

Instrumentation time was lower for the TN group a median and range 14.5 (7—42) seconds (sec.) in comparison to the RC group which recorded a median and range of 35 (10 – 47) sec. The mean time was 17.7 (9.9) sec. for the TN group, and 30.3(13) Sec for the RC. There was a significant difference between the two groups (*p* = 0.003) (Fig. 8).

**Fig. 9:**
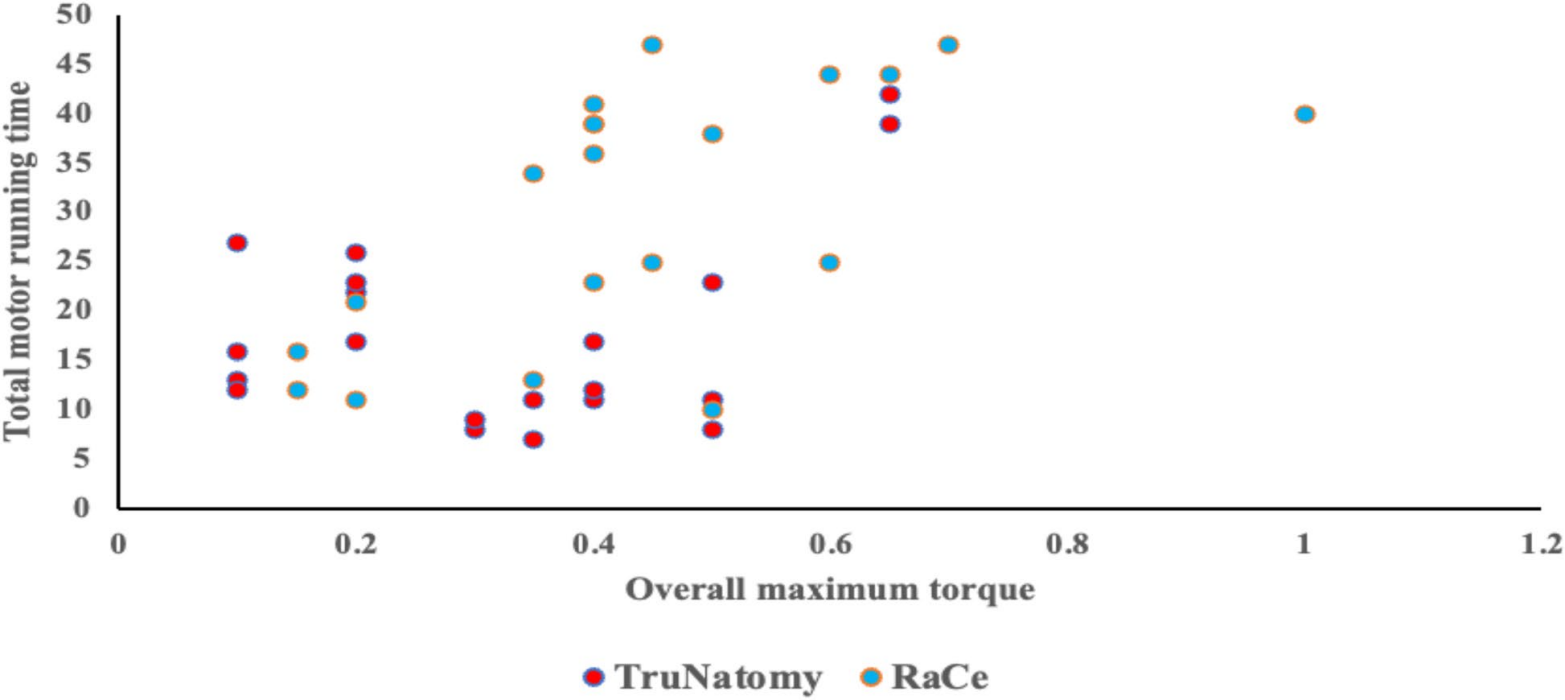
Scatter plot between the maximum torque and total motor running time

The relationship between the overall maximum torque and the whole motor running time was investigated using Spearman rank-order correlation. The two variables had a moderately significant positive connection (rs = 0.463, *n* = 40, *p* = 0.003). (Fig. 9).

## Discussion

The degree of post operative pain has a direct impact on how the patient evaluates the dentist's abilities. Both the patient's quality of life and the dentist-patient relationship may be negatively or positively impacted by the postoperative experience [14, 15]. Accordingly, the incidence and reduction of post-endodontic pain and flare-ups have constantly remained a topic of interest and a fundamental goal for endodontic treatment.

The operative torque generated during use and the time needed for the instrument to reach the WL can be used as an indicator of instrument's cutting efficiency, expected durability, and overall safety [8]. This testing method has the advantage of more accurate replication of the clinical conditions where instruments are subjected to an assimilation of forces distributed across their entire length simultaneously [9]..

Bi-rooted maxillary premolars were selected for the study owing their tight canals sizes with their minor apical constriction diameter being reported to be as small as 0.1 mm [16]. This was thought to allow for emphasis to be placed on the instruments cutting efficiency and concurrently its generated operative torque levels. Additionally, A large percentage of these teeth are characterized by highly concave furcation groove that can act as a danger zone after endodontic treatment serving as nuclei for crack propagation and the initiation of vertical root fracture [17]. Therefore, a more conservative preparation strategy such as the one proposed by the TN rotary system, emphasizing dentin preservation in these areas may be of special benefit for these teeth.

Asymptomatic teeth necrotic teeth were chosen to allow for the exclusion of pre-operative pain as a predicting factor, which is mostly agreed upon to be the prime factor influencing the incidence and severity of postoperative pain [18]. In asymptomatic necrotic teeth, factors such as the amount of debris extrusion, the virulence of microorganisms, and host defence play a more pivotal role in the patient's post-operative pain experience [19].

Teeth with periapical lesions were excluded from the study as they may represent a sign of long-standing infection, cystic transformation, or extra-radicular infection which may negatively influence the treatment outcome [20]. Furthermore, apical resorption and disruption of the apical constrictions are common occurrences in teeth with necrotic pulps and large periapical lesions. This in turn may influence the degree of extrusion of debris and irrigant from the apical foramen and potentially complicate the root canal treatment procedure [21].

Only the first instrument of each sequence was utilized to assess the operative torque and instrumentation time. This choice was made as previous studies have demonstrated that the smaller the canals the higher the torque required to remove dentin and progress into the full working length [22]. Teeth with both canals larger than size #15 were excluded, allowing standardization of the initial canal size as much as feasible, leaving the effect of instrument design as the main factor influencing the generated torque. The subsequent instruments for both systems were excluded from the analysis as it was thought that the larger size and taper for subsequent instruments in RC instruments would inherently result in more torque generation, thus not accurately reflect the impact of instrument design [23].

Instruments were used at the manufacturer-recommended speeds (TN: 500 RPM; RC: 600 RPM) to reflect real-world clinical use. While changing the instrument speed may have an impact on the amount of torque generated, testing at recommended speeds ensured clinically relevant performance data while maintaining each system's intended cutting efficiency and safety profile [24].

A mean effect size of (d = 1) was utilized for our sample size calculation, this aligns with established clinical thresholds for meaningful differences for VAS pain diary which places the minimum clinically significant VAS difference between 0.9–1.3 [25, 26].This prioritized the detection of clinically impactful pain reductions over statistical significance of minor variations that may not influence treatment decisions.

The lack of significant difference in the pain incidence and intensity between the two groups could be explained by the design feature of both systems TN off-centered parallelogram slender cross-section, its regressive taper, and the variable short pitch length may have allowed for more space for the coronal evacuation of necrotic debris during the instrumentation process leading to less apical debris extrusion [27]. Likewise, the small core size, non-convex triangular cross-sectional design, and alternating helical angle of the RC instruments may have also played a similar role in providing a space for coronal debris evacuation and limiting apical extrusion of debris [28].

To our knowledge, no previous study utilizing TN rotary system has attempted to examine post-operative pain in asymptomatic necrotic teeth. Studies examining post-operative pain following the use of TN on vital teeth showed varying results. Lower incidence of post-operative pain was seen for TN files when compared to TF and TFA files [29]. On the other hand, two studies found it to have higher incidence of pain when compared to other contemporary rotary and reciprocating files [30, 31].However, the increased pain for the TN group in these situations can be attributed to either incomplete shaping of the canals only up to TN’s Prime files size (#26 variable taper 0.04) or utilization of fewer number of instruments in the comparator group.

The highest reported pain scores were observed at 6-hour marks, followed by a steady decline in pain scores where at the one-week mark no pain was reported for both groups. This could be explained by the fading effect of the local anesthetic agent which may have coincided with the transient local inflammatory response induced by the irritation of the periapical area by the extruded bacteria and necrotic debris during the endodontic treatment. This, in turn, could provide a potential explanation for the relatively high initial pain scores observed at the 6-hour mark. The gradual decline in pain scores following this point can be credited to the recovery of the periapical area [32].

Overall, the range of pain scores for all groups was low not exceeding 3.0 cm out of 10cm on the modified VAS scale, which was highlighted by a previous study to be within what is known as the patient-acceptable symptom status (PASS) [33]. The low pain levels recorded were reflected in the lack of use for the rescue analgesics medication in both groups.

Although there was no statistically significant difference in Mean operative torque, average peak torque, and maximum torque between the groups, TN rotary instruments showed a trend toward consistently generating lower mean torque values. This again can be credited to the previously mentioned design features. TN’s slim wire, regressive taper, and off-centered cross section are professed by the manufacturer to decrease the torque acting on the instrument through decreasing the contact area between instrument and root canal subsequently decreasing the friction and allowing for better stress distribution and less stress concentration. Furthermore, these same features may have positively impacted coronal debris evacuation and subsequently the cutting efficiency and in turn the generated operative torque. Additionally, the predominantly martensite alloy of the TN system may have positively impacted the instrument cutting efficiency and subsequently the resultant operative torque when compared to RC instruments [34].

No previous clinical study has attempted to examine real-time torque parameter of the TN rotary files in a clinical setting. However, Previous in-vitro studies have enforced TN’s shaping files ability to reduce torque generation and threading forces during instrumentation [35, 36].

The working time was only recorded from the time of the instrument insertion and up to the point where the instrument reached the full WL with only active instrumentation time being taken into consideration. This was done to avoid confounders that can be introduced by including the instrument change time, irrigation time and other factors that may widely vary between different operators.

Similar to the operative torque parameter the previously mentioned design features were the prime factor controlling the instrumentation time. The operative torque and instrumentation time are complementary aspects which influence each other; accordingly, their results should not be interpreted as separate outcomes as both serve as co-markers for the overall cutting efficiency, durability, and safety profile of the instrument [23]. RC initial instruments demonstrated statistically significantly longer instrumentation time in comparison to corresponding TN instruments and took on average 12.6 secs more to reach the working length. This indicates that shorter amplitude strokes were used with each pecking motion leading to slower rate of advancement inside the canals. Despite that RC instruments still produced higher levels of operative torque indicating more difficulties in advancing the instrument to the WL when compared to the corresponding TN instrument.

While a mean difference of 12–13 secs may initially seem clinically insignificant. The potential synergetic effect of how this number may add over the full instrumentation sequence over multiple canals and over multiple cases in a busy work environment may present substantial advantage in the clinical setting especially if supplemented with other time saving approaches relating to the treatment procedure. This in turn could have a significant impact on both the clinician comfort and patient’s satisfaction.

Interpretation of the results of both time and torque as one would again indicate that the TN rotary files could advance faster, with fewer numbers of strokes needed while generating relatively lower levels of torque. Moreover, previous studies examining the dynamic torsional resistance of RC and TN have found that the mean dynamic torsional resistance of the TN instruments was roughly five time higher than that of the RC instruments utilizing the exact same methodology [37, 38]. This in turn imply that TN instruments can withstand a higher level of repeated stresses in comparison to the RC instruments. Thus, a rationale composite interpretation for these results would imply higher cutting efficacy and safety profile for TN rotary system during clinical use even at comparable torque levels in comparison to the RC rotary system.

Previous in-vitro studies utilizing the same method for measuring the working time of TN have shown similar results with TN consistently having the shortest instrumentation in comparison to other rotary and reciprocating systems [39, 40]

A significant positive moderate correlation was found between the overall maximum torque and total motor running time. This is understandable as the higher torque values represents the difficulty in advancing the instrument through the canals. Accordingly, the harder it’s for the instrument to advance through the canal, the more time it would take for it to finally reach the WL. The positive correlation between high torque values and time needed to reach the WL was confirmed in a previous systematic review [23].

To our knowledge this was the first randomized clinical trial to investigate the parameter of operative torque of the TruNatomy rotary files in a standardized clinical setting. While strict inclusion criteria and standardized protocol may adversely affect the generalizability, they ensure a homogeneous sample and reduce confounding variables, enhancing the internal validity of the findings.

While this study provides valuable insights into the efficacy and safety of TN and RC rotary systems, limitations such as being a single-center design and small sample size as well as the strict inclusion criteria may limit the generalizability of our findings. Additionally, having all the procedures performed by a single operator may also introduce operator specific biases. Furthermore, the finding of the study may not extend to teeth with moderate to severe canal curvatures. It should also be noted that our results for operative torque primarily reflect the initial instrument of each system, larger files in the RC sequence would likely exhibit even greater torque generation due to its larger tip size and taper in comparison to the corresponding TN instrument.

While our study was adequately powered to detect clinically meaningful differences in pain the possibility of Type II error cannot be excluded for smaller effect sizes where larger samples sizes may be warranted.

Future research is needed to validate the parameter of operative torque, as well as utilizing its data for the torque range parameter in larger population and in more diverse and complex clinical settings.

## Conclusion

Within the limitation of this study, it could be concluded that TN and RC resulted in similar and acceptable levels of post-operative pain in cases of asymptomatic necrotic teeth with no significant difference between both instruments. While the lower torque values of TN instruments were not statistically significant, combined interpretation of the torque trends and time results would indicate the TN instruments were able to advance inside the root canals significantly faster during initial instrumentation with potentially less stress on the instrument, suggesting a superior performance, cutting efficiency, and durability of the instruments tested which is likely to persist throughout the instrumentation sequence. These characteristics may enhance the procedural efficiency and reduce the time required for treatment, potentially improving patients’ satisfaction.

## Data Availability

The data sets for the current trial are available at clinical trial.gov under the identifier NCT04616469.The authors confirm that the data supporting the findings of this study are available within the article and its Supplementary material. Raw data that support the findings of this study are available from the corresponding author, upon reasonable request.
